# PTSD is not the emblematic disorder of the COVID-19 pandemic; adjustment disorder is

**DOI:** 10.1186/s12888-022-03903-5

**Published:** 2022-04-28

**Authors:** Alain Brunet, Marjolaine Rivest-Beauregard, Michelle Lonergan, Sabrina Cipolletta, Andrew Rasmussen, Xiangfei Meng, Nematollah Jaafari, Sara Romero, Julia Superka, Adam D. Brown, Ram P. Sapkota

**Affiliations:** 1 Research Center of the Douglas Mental Health University Institute (CIUSSS-ODIM), 6875 boulevard LaSalle, Montreal, QC H4H 1R3 Canada; 2 Department of Psychiatry, McGill University, Montreal, QC Canada; 3 School of Psychology, Ottawa University, Ottawa, ON Canada; 4 Department of General Psychology, University of Padua, Padua, Italy; 5 Department of Psychology, Fordham University, New York, NY USA; 6 Department of Psychiatry, Université de Poitiers, Poitiers, France; 7 Department of Global Health and Social Medicine, Harvard Medical School, Boston, MA USA; 8 Department of Psychology, The New School for Social Research, New York, NY USA; 9 Online Therapy Unit, Department of Psychology, University of Regina, Regina, SK Canada

**Keywords:** Post-traumatic stress, Adjustment disorder, COVID-19, Coronavirus, Resilience

## Abstract

**Background:**

Posttraumatic stress disorder (PTSD) has been hailed by some as the emblematic mental disorder of the COVID-19 pandemic, assuming that PTSD’s life-threat criterion was met de facto. More plausible outcomes like adjustment disorder (AD) have been overlooked.

**Methods:**

An online cross-sectional survey was launched in the initial stage of the pandemic using a convenience sample of 5 913 adults to compare the prevalence of COVID-related probable PTSD versus probable AD. The abridged Impact of Event Scale – Revised (IES-6) assessed the severity of trauma- and stressor-related symptoms over the previous week. Demographic and pandemic-related data (e.g., receiving a formal diagnosis of COVID-19, job loss, loss of loved one, confinement, material hardship) were collected. A Classification and Regression Tree analysis was conducted to uncover the pandemic experiences leading to clinical ‘caseness’. Caseness was defined by a score > 9 on the IES-6 symptom measure and further characterized as PTSD or AD depending on whether the Peritraumatic Distress Inventory’s life-threat item was endorsed or not.

**Results:**

The participants were predominantly Caucasian (72.8%), women (79.2%), with a university degree (85%), and a mean age of 42.22 (*SD* = 15.24) years; 3 647 participants (61.7%; 95%CI [60.4, 63.0]) met the threshold for caseness. However, when perceived life-threat was accounted for, only 6.7% (95%CI [6.1, 7.4]) were classified as PTSD cases, and 55% (95%CI [53.7, 56.2]) as AD cases. Among the AD cases, three distinct profiles emerged marked by the following: (i) a worst personal pandemic experience eliciting intense fear, helplessness or horror (in the absence, however, of any life-threat), (ii) a pandemic experience eliciting sadness/grief, and (iii) worrying intensely about the safety of significant others.

**Conclusions:**

Studies considering the life-threat criterion as met de facto during the pandemic are confusing PTSD for AD on most counts. This misconception is obscuring the various AD-related idioms of distress that have emerged during the pandemic and the actual treatment needs.

## Introduction

Several research teams across the world have been sounding the alarm about the deleterious effects of the COVID-19 pandemic on mental health (e.g., [1–3]). Some groups have published extremely high rates of posttraumatic stress symptoms and disorder (PTSD; [4–7]) raising deep concerns with respect to the population’s mental health. However, certain criteria must be met in establishing a diagnosis of PTSD. Among these is the sound identification of a life-threat [8, 9]. This criterion is often overlooked in PTSD research involving self-report inventories, and with respect to the COVID-19 pandemic it has been considered as met de facto. For instance, although one study [6] did use a measure of trauma exposure, the authors did not consider the life-threat requirement in their computation of PTSD prevalence. Other studies [4, 5, 7] relied solely on cut-off scores from self-report measures of PTSD symptoms to determine prevalence estimate. In this study, we aimed to examine the prevalence of PTSD ‘caseness’ (i.e., meeting enough criteria to be classified as a case for a disorder) during the pandemic compared to other, less frequently mentioned stressor-related disorders like adjustment disorder (AD), not taking the life-threat criterion for granted.

AD is the *forme fruste* of PTSD; the more mundane diagnostic entity of the DSM-5’s *Trauma- and stressor-related disorders* category [10]. It is the personal tragedy without the life-threat: the job losses, the worrying about significant others, the catastrophic financial losses, the prolonged separations, the disputes and divorce, or the painful grief of having lost a loved one [8, 11–13]. Importantly, the symptom profiles of AD and PTSD overlap substantially. Thus, we wondered if the pandemic-related stress-response syndromes would be better captured by the polymorphous diagnosis of AD, a common but neglected disorder [10] for which professionals lack guidance on the treatment guidelines [14].

### Rethinking the so-called PTSD cases

As part of an online international survey, we operationalized probable AD and probable PTSD ‘caseness’ (henceforth simply called AD and PTSD) based on information drawn from two well-established event-related self-report symptom measures: the Peritraumatic Distress Inventory and the abridged Impact of Event Scale – Revised [15, 16]. We hypothesized that, once properly classified, AD would be more prevalent than PTSD among adults disclosing the mental health impact of their worst pandemic experience. Using an inductive classification and regression tree analysis, we also explored the psychosocial determinants of caseness beyond life-threat to uncover some idioms of distress emerging from the adversity induced specifically by the pandemic.

## Methods

### Sampling, procedure and survey design

Ethics approval (#IUSMD-20–13) was first obtained from the *Centre Intégré Universitaire de Santé et de Services Sociaux Ouest-de-l'Île-de-Montréal* (CIUSSS-ODIM) Research Ethics Board, Douglas Mental Health University Institute (Montreal, Canada) for all the selected countries. A convenience sample of 5 913 adults from Italy, Canada, the United States, France, and China was surveyed cross-sectionally between 04/19/2020 and 05/26/2020. Note that at the time of data collection, all countries of interest except for China were under emergency alert, with country borders closed to non-residents, strict lockdowns, and stay-home orders implemented. China which had been under national quarantine earlier was starting its deconfinement process (see newspaper articles of the NY Times and The Guardian). Recruitment was conducted via online advertisements and email invitations sent to local academic and non-academic individuals and associations (e.g., university alumni, health workers, psychologists) using the snowball technique. Survey completion among those who opened the survey’s hyperlink was 92%. Participants provided informed consent and completed the 15-min self-report survey on the *SurveyMonkey* web platform (click here to review their privacy statement) in one sitting. Sample characteristics are presented in Table 1.

**Table 1:** Sociodemographic and clinical variables of the sample (*N* = 5 913)

	*M*	*SD*
Age (years)	42.22	15.24
Peritraumatic distress^1^	17.53	10.56
Trauma- and stressor-related symptoms^2^	11.24	5.85
	*N*	%
Country of Residence		
France	1 036	17.52
Canada	1 946	32.91
Italy	1 094	18.50
USA	1 302	22.02
China	336	5.68
Other	199	3.37
Gender		
Man	1 173	19.84
Woman	4 681	79.16
Other/ won’t disclose	59	1.00
Marital Status		
Single	2 156	36.46
Cohabitating/ married	3 278	55.43
Separated/ Divorced/ Widowed	479	8.10
Ethnicity		
Caucasian	4 306	72.82
Black	68	1.15
Hispanic	316	5.34
Asian	617	10.43
Mixed	123	2.08
Other	483	8.17
Occupation^3^		
Stay home occupations	676	11.43
Essential workers	2 717	45.95
Non-essential workers	1 578	26.69
Other	942	15.93
Education		
Pre-University	837	14.16
University degree (undergraduate)	2 064	34.91
University degree (graduate)	3 012	50.94

^1^Peritraumatic Distress Inventory

^2^Abridged Impact of Event Scale – Revised (IES-6)

^3^Occupation categories were as follows. Stay home occupation: unemployed, homemaker, and retired. Essential workers: manual workers, professionals, defined as employments requiring university-level education and expertise (e.g., health workers, school directors, teachers, researchers, etc.), and military. Non-essential workers: students, and non-essential retail/trade workers/business owners

### Instruments and operationalization of caseness

The Peritraumatic Distress Inventory (PDI) [15] assessed life-threat and 12 other physical and emotional responses (e.g., I was horrified by what I saw, I felt helpless to do more) experienced with respect to the most difficult event occurring during the pandemic, on a scale from 0 (not at all) to 4 (extremely true). For data analytic purposes, the PDI items were dichotomized as present or absent using a score of 3 (very true) or 4.

Event-related distress over the past 7 days was assessed with the abridged Impact of Event Scale – Revised [16, 17] (IES-6) with respect to the same ‘worst’ event identified on the PDI. The six items of the scale, rated 0 (not true) to 4 (extremely true), measure the transdiagnostic symptoms of intrusions, avoidance, and hyperarousal observed across all trauma- and stressor-related disorders, including PTSD, AD, and grief reactions (α = 0.85). A total score is obtained by summing all six items (range 0–24). Thoresen et al. [16] proposed a clinical cutoff score of 10.

**Fig. 1:**
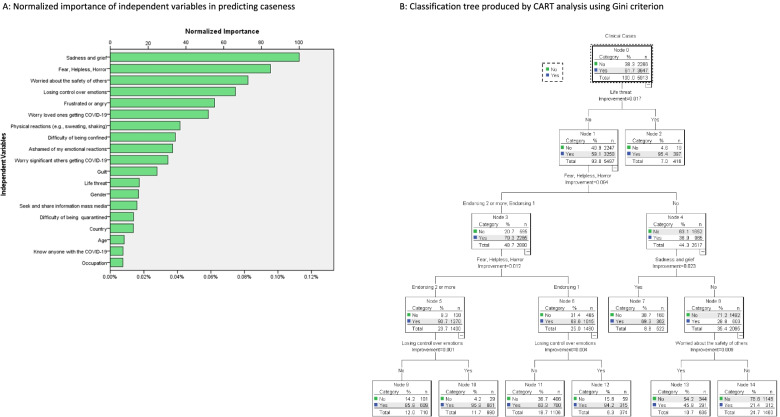
**A** Normalized importance of independent variables in predicting caseness. **B** Classification tree produced by CART analysis using Gini criterion. Node 0 contains frequency counts and percentages of all observations in the model on the dependent variable, IES-6. Nodes 1–14 display the number and percentage of participants in that subgroup and the percentage of accurate classification of caseness by that subgroup. Dependent variable: Impact of Event Scale (IES-6). Independent variables: Life threat, Fear, Helpless, Horror, Sadness and grief, Worried about the safety of others, Frustrated or angry, Worry loved ones getting COVID-19, Worry significant others getting COVID-19, Losing control over emotions, Difficulty of being confined, Country, Physical reactions (e.g. sweating, shaking), Gender, Seek and share information mass media, Guilt, Know anyone with the COVID-19, Ashamed of my emotional reactions, Occupation, Difficulty of being quarantined, Connection to and support social media, Age, Ethnicity, Number of children, Level of education, Pregnancy status, Separated from loved ones, Socially isolated, Professional emotional help & support, Quarantined, Family emotional help & support, Friend emotional help & support, Experienced COVID-19 symptoms, Felt I might pass out, Exposed to infected individuals or objects as part of work, Difficulty controlling bowel and bladder, Received financial support, Experienced material hardship, Tested for the COVID-19, Travel in or out of your home country, Significant other experienced COVID-19 symptoms, Lost loved ones, Lost your job, At-risk group for the COVID-19, Diagnosed with the COVID-19, Marital status, Received medical care for COVID-19, Hospitalized for COVID-19, Significant other diagnosed with the COVID-19, Been at ICU for COVID-19

PTSD ‘caseness’ (i.e., being a clinical case) was defined as individuals scoring 10 or more on the IES-6 and endorsing a rating of 3 (very true) or 4 on item 13 of the PDI (*I thought I might die*). Individuals scoring above the IES-6 cut-off but who did *not* endorse the life-threat item (a.k.a. PTSD’s trauma exposure criterion) were considered as cases of AD rather than PTSD. The 12 other PDI items, the sociodemographic and other COVID-related variables (see Fig. 1A and B) served as psychosocial determinants of caseness.

### Sample size and statistical analyses

Of the 6 409 initial respondents, 496 were removed for not completing any part of the survey, resulting in a final sample of 5 913. Little’s MCAR test [18] suggested that the missing data (5%) were not missing completely at random, χ^2^ = 15,480.5, *df* = 13,558, *p* < 0.001. Data were therefore imputed using the *k*-Nearest-Neighbor imputation method with *k* = 5 in the VIM package for the statistical software R [19, 20]. All other analyses were performed using SPSS v23 (IBM Corp., Armonk, NY).

Classification and regression tree (CART; [21]) is an inductive data analytic method that uses recursive partitioning to split a sample into mutually exclusive subgroups. CART can uncover interactions in subgroups that are not otherwise discoverable with traditional regression methods. In addition, in CART, the importance of each independent variable is considered in isolation to the overall model [21, 22]. To avoid over-fitting the decision tree, the cases required in the terminal node was set to 250 (roughly 5% of the sample) and node splitting was stopped when Gini < 0.001 [22]. To assess the model’s stability, the data were randomly divided into 10 subsets and the classification produced with 90% of the data was applied to the remaining 10% (see [23]). To differentiate PTSD from AD cases, the life-threat variable of the PDI was forced as the first splitting variable in the CART model.

## Results

### Sample characteristics

The sample, as per Table 1, was predominantly Caucasian, female, and educated; 1 080 participants (18.3%) experienced some COVID-19 symptoms while 149 (2.5%) received a formal medical diagnosis. In addition, 167 (2.8%) participants lost a loved one because of COVID-19. The PDI cutoff score of 14, which indicates clinical levels of event-based (peritraumatic) distress [24], was met by 3 336 participants (56.4%).

### CART model performance

The accuracy achieved by the CART model was 76.7%. Similar classification accuracy was obtained when variables appearing in the CART model were fitted in a logistic regression. There was no difference in the risk estimate (23%) in the tenfold cross-validation model, suggesting that the model was stable.

### Rates of AD and of PTSD

The number of participants meeting or exceeding the IES-6 clinical threshold was 3 647 out of 5 913, yielding a global caseness rate of 61.7% (95%CI [60.4, 63.0]), as seen in Fig. 1B. Cases were then partitioned into those reporting a life-threat (6.7%; 95%CI [6.1, 7.4]) and those who didn’t (55%; 95%CI [53.7, 56.2]). The former were considered as PTSD cases while the later were considered as AD cases. Non-cases amounted to 38.3% (95%CI [37.1, 39.6]).

### Psychosocial determinants of AD and PTSD caseness

As shown in node 1 of Fig. 1B, of the 416 participants endorsing the PDI’s life-threat item, 397 (95.4%) reported symptomatic distress above the clinical threshold, highlighting the very potent predictive power of life-threat for developing PTSD. No other variable enhanced the prediction of PTSD caseness.

The data were further partitioned to uncover the psychosocial determinants of AD. Peritraumatic fear, or helplessness or horror in response to the worst COVID-related event led to AD caseness with a probability of 79.3% (node 3). AD was further predicted by endorsing two or all three of the abovementioned peritraumatic reactions (90.7%; node 5), rather than only one of the three, and even more so by further endorsing feelings of having lost control over one’s emotions (95.8%; node 10). In sum, this branch of the tree outlined a path to AD caseness consisting of intense pandemic-related peritraumatic distress (fear, helplessness, horror, etc.), but in the absence of a personal life-threat.

In the absence of life-threat, and in the absence of fear, helplessness, or horror, the endorsement of the PDI’s peritraumatic sadness and grief item led to AD caseness in 69.3% of such participants (node 7). Finally, in the absence of experiencing life-threat, in the absence of fear, helplessness, or horror, and in the absence of sadness and grief, worrying intensely about the safety of others led to AD caseness in 45.8% of participants reporting it (node 13).

Altogether, those results suggest a 5-pronged typology of COVID-related distress. (i) A first subgroup experienced a life-threat and met the PTSD criteria. Among the AD cases, three profiles were found: (ii) trauma-like symptoms triggered by experiencing intense fear, helplessness or horror, but in the absence of a life-threat, (iii) sadness and grief reactions and (iv) intense worrying toward the safety of other. (v) A fifth group was composed of resilient individuals not affected by adversity or simply failing to endorse enough symptoms to become a clinical case.

### COVID-19 diagnosis and perceived life-threat

To further explore whether the life-threat criterion should be considered as met de facto during the COVID-19 pandemic, we tested whether receiving a formal COVID-19 diagnosis was associated with reporting a life-threating experience. A Chi-square test indicated a statistically significant (due to an excess of statistical power), although very weak (φ = 0.10), association between perceived life-threat and being diagnosed with the COVID-19 (see Table 2).

**Table 2:** Counts and proportions of COVID-19 diagnoses and Perceived life-threat during the pandemic in the sample (*N* = 5 913)

	Were you officially diagnosed with the COVID-19?		
Perceived life-threat	No	Yes	Total
No	5 383 (91.0)	114 (1.9)	5 497 (93.0)
Yes	381 (6.4)	35 (0.6)	416 (7.0)
Total	5 764 (97.5)	149 (2.5)	5 913 (100)

Cells (*n*, *%*). χ^2^(1) = 63.28, *p* < .001

The association between perceived life-threat and being diagnosed with the COVID-19 is weak, with φ = 0.10, *p* < .001

## Discussion

The current study used a cross-sectional survey sample of COVID -19 pandemic-exposed adults living in five “most hardly” hit countries – China, the United States of America, France, Italy, and Canada – to determine the prevalence of Trauma- and Stressor-related disorders.

The overall proportion of participants meeting the clinical threshold for PTSD was 6.7% and 55% for AD, suggesting that AD is by far the more prevalent form of distress experienced during the COVID-19 pandemic, not PTSD. This was the case for all five countries surveyed in this study (results not shown for conciseness).

Remarkably, we found that a single question, the PDI’s life-threat item, captured 95% of the PTSD cases, a very robust finding that concurs with the published literature [25]. However, reporting this experience was not as common as one would think, with less than 7% of the sample endorsing it. It is therefore inappropriate to consider the life-threat criterion as met universally during the pandemic. Even the association between receiving a COVID-19 diagnosis and feeling that one’s life had been threatened was found to be rather weak in this sample. Although several recent studies have reported alarming rates of posttraumatic stress, the experience of life-threat was usually inferred from either a positive COVID-19 diagnosis or by being exposed to the pandemic per se (see [4–6]). These studies, therefore, have inflated the true rate of PTSD, clouding the fact that AD is more prevalent than PTSD. The assessment of PTSD without evaluating the life-threat trauma criterion is not methodologically sound, and such practices should be discontinued due to the risk of disseminating misleading information to the public, the treatment providers and the policy planners.

Instead of blindly classifying distressed cases as PTSD, we found three mutually exclusive AD profiles. The first and largest AD group experienced at least one pandemic event eliciting ‘trauma-like’ peritraumatic responses of intense fear, helplessness, and horror but in the absence of any personal life-threat. It is interesting to note that in the 5^th^ revision of the DSM, the subjective dimension of trauma exposure (the so-called PTSD criterion A2 from DSM-IV, which consisted of fear, helplessness, and horror reactions) was abandoned, allegedly for lack of predictive power [26]. However, in life situations where the life-threat is diffuse or ambiguous, the reporting of such experiences identified many clinically distressed individuals. A second group of distressed individuals experienced intense sadness/grief as part of their worst experience of the COVID-19, which suggests the presence of a depressive-like reaction related to some form of personal loss. A third group worried primarily and intensely about the safety of others during the pandemic, to the point of becoming clinically distressed themselves.

Such findings provide further evidence for AD as a polymorphous event-based stress-response syndrome, not unlike PTSD or prolonged grief [27]; (see also [28, 29]). It is time to acknowledge that in the aftermath of most disasters or most tragedies, a small but important number of individuals will develop PTSD; however, an equally important group, and more numerous groups, of individuals will develop an AD, not PTSD.

There is currently no published placebo-controlled evidence-based treatment for AD, representing an important knowledge gap. It is time to stop confusing AD for PTSD. AD is the elephant in the room. It needs to be acknowledged as an important outcome in the aftermath of disasters and be taken seriously as a full-fledged diagnostic entity.

This study has several limitations. The rates of AD and PTSD cases reported here were obtained in an inherently biased convenience sample. Although the rates of AD and PTSD may vary depending on the sampling strategy employed, the ratio of AD to PTSD in each of the five countries that we surveyed was similar and always largely in favor of AD. This suggests that our finding is a robust one, which would be replicated in an epidemiological study using an unbiased (random) sampling strategy. The term probable AD or PTSD was used in this report, to remind the reader that a psychiatric diagnosis cannot be inferred using self-report measures and cut-off scores [30].

## Conclusions

The intention behind this study was to illustrate that once the experience of perceived life-threat is measured rather than taken for granted, AD is much more common than PTSD during the COVID-19 pandemic and will likely exert its consequences on mental wellness across the globe for the months and years to come. CART analyses suggested the presence of three different forms of AD which may require different treatment approaches. Unfortunately, there is currently no evidence-based treatment for AD, representing a knowledge gap that requires urgent attention.

## Data Availability

The datasets generated and/or analysed during the current study are not publicly available but are available from the corresponding author on reasonable request.
